# LncRNA ADAMTS9-AS1 knockdown suppresses cell proliferation and migration in glioma through downregulating Wnt/β-catenin signaling pathway

**DOI:** 10.17305/bjbms.2021.6199

**Published:** 2021-12-11

**Authors:** Chunhui Zhou, Hulin Zhao, Shuiwei Wang, Chao Dong, Fan Yang, Jianning Zhang

**Affiliations:** Department of Neurosurgery, Medical School of Chinese PLA General Hospital, Beijing, China

**Keywords:** lncRNA, ADAMTS9-AS1, glioma, prognosis, Wnt/β-catenin

## Abstract

The long non-coding RNA antisense 1 ADAMTS9-AS1 has been reported to serve as an oncogene or tumor suppressor in several tumors, including colorectal cancer and hepatocellular carcinoma. Nevertheless, the clinical significance and biological behaviors of ADAMTS9-AS1 in glioma still remain unclear. Therefore, the goal of this study was to evaluate the functional roles and potential mechanisms of ADAMTS9-AS1 in glioma cells. Using quantitative real-time polymerase chain reaction analysis, we found that ADAMTS9-AS1 was upregulated in glioma tissues and cells in comparison to corresponding controls. ADAMTS9-AS1 expression level was correlated to tumor size (*p* = 0.005) and the World Health Organization grade (*p* = 0.002). Kaplan–Meier analysis and Cox multivariate analysis showed that ADAMTS9-AS1 could serve as an independent prognostic factor affecting the overall survival of glioma patients. Functionally, depletion of ADAMTS9-AS1 significantly suppressed the proliferation, migration, and invasion in glioma cell lines (U251 and U87), as shown through cell counting kit-8 assay, Edu corporation assay, wound healing assay, and transwell assay. Furthermore, we demonstrated that knockdown of ADAMTS9-AS1 suppressed Wnt1, β-catenin, c-myc, and proliferating cell nuclear antigen, while upregulating E-cadherin expression. In conclusion, our data revealed that ADAMTS9-AS1 confers oncogenic function in the progression of glioma, thus targeting ADAMTS9-AS1 might be a promising therapeutic strategy for this disease.

## INTRODUCTION

Glioma is considered to be the most common primary intracranial tumor type with high mortality and morbidity [1]. According to the 2016 World Health Organization (WHO) classification, glioma could be subdivided into low grade (WHO Grades I and II) and high grade (WHO Grades III and IV) [2]. To the best of our knowledge, Grade I tumors are generally benign and frequently curable with complete surgical resection [3]. However, the prognosis of glioma patients from Grades II-IV under chemotherapy and target therapy still remains poor, with median survival time of approximately 14 months [4-6]. Therefore, it is significantly essential to explore the molecular mechanisms that affect the malignant processes of glioma.

Long non-coding RNAs (lncRNAs) are a group of non-protein-coding RNAs with more than 200 nucleotides in length, which regulate various biological functions, including cell proliferation, migration, and invasion associated with glioma [7,8]. For instance, Zhang et al. [9] revealed that lncRNA ASB16-AS1 exhibited higher expression levels in glioma tissue samples and its knockdown inhibited the proliferation, invasion, and migration of glioblastoma stem-like cells. LncRNA HOXA11-AS silencing could inhibit cell migration, invasion, proliferation, and promote apoptosis in U251 cells [10]. On contrast, Hu et al. [11] reported that lncRNA PLAC2 overexpression inhibited glioma cell growth and induced cell cycle arrest in a (STAT)1/RPL36-dependent manner *in vitro* and *in vivo*. In addition, tumor suppressors, including CASC7 [12] and PTENP1 [13], as well as oncogenes, including SNHG16 [14] and PVT1 [15], play essential roles in glioma. However, investigation of these reported molecular mechanisms is far from enough and identification of additional candidate biomarkers is still needed.

In recent years, lncRNA ADAM metallopeptidase with thrombospondin type 1 motif, 9 antisense RNA 1 (ADAMTS9-AS1), located on chromosome 3p14.1, was of special interest in carcinogenesis research. Bioinformatics analysis identified that ADAMTS9-AS1 participated in the initiation and progression of epithelial ovarian cancer [16], and demonstrated a role of a novel prognostic biomarker in esophageal squamous cell carcinoma [17], papillary renal cell carcinoma [18], breast cancer [19], and bladder cancer [20,21]. In addition, Wan et al. [22] found that ADAMTS9-AS1 significantly influenced tumor cell growth and proliferation, suggesting that it plays a tumor-suppressive role in prostate cancer. Moreover, Zhang et al. [23] demonstrated that ADAMTS9-AS1 contributed to proliferation, migration, and invasion in hepatocellular carcinoma cells, likely due to the activation of the PI3K/AKT/mTOR signaling pathway. Depletion of ADAMTS9-AS1 significantly suppressed cell proliferation, G1/S transition, migration, and invasion in colorectal cancer [24]. On the other hand, Li et al. [25] revealed that ADAMTS9-AS1 suppressed the colorectal cancer cell proliferation and migration by inhibiting the Wnt/β-catenin signaling pathway. Nevertheless, the biological functions of ADAMTS9-AS1 in glioma cells have not been reported yet. These observations suggest that ADAMTS9-AS1 may be a crucial regulator involved in the biological functions of glioma cells.

To validate our hypothesis, we evaluated the functional roles and potential mechanisms of ADAMTS9-AS1 in glioma cells. We not only investigated the clinical significance of ADAMTS9-AS1 as a prognostic marker in glioma patients but also assessed the effects of ADAMTS9-AS1 on glioma cell proliferation, migration, and invasion based on a group of *in vitro* assays. Furthermore, we investigated the underlying mechanisms of ADAMTS9-AS1 on glioma cells.

## MATERIALS AND METHODS

### Patients and tissue samples

Human tumor tissue samples were collected from 79 glioma patients undergoing an initial surgery at the Medical School of Chinese PLA General Hospital during the period from 2015 to 2018. All the patient samples were diagnosed pathological and confirmed by a local pathologist. The basic clinicopathological features, including age, gender, the WHO grade, and follow-up information, are summarized in Table 1. According to the 2016 WHO classification, 37 cases were Grades I–II and 42 were Grades III–IV. In addition, normal brain tissues were obtained from 20 patients undergoing brain tissue resection for craniocerebral injury. All enrolled patients signed the written informed consent. All collected tissue samples were preserved in liquid nitrogen. This study was approved by the Human Research Ethics Committee of Medical School of Chinese PLA General Hospital (Approval no. MSCP-G237, 2018.4.22, Shandong Province, China).

**TABLE 1:**
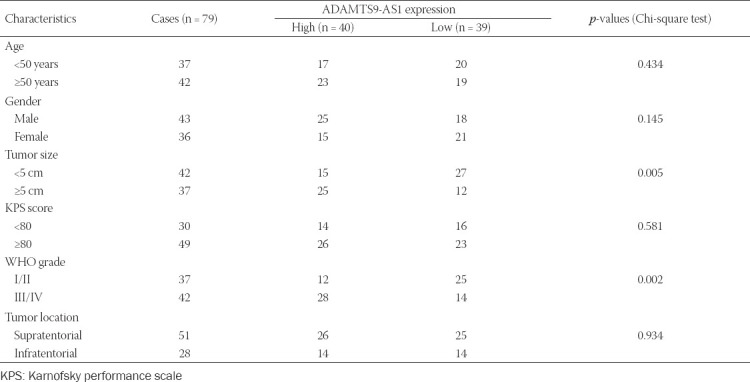
Association between ADAMTS9-AS1 expression and clinicopathological characteristics of glioma patients

### Cell culture and transfection

Human glioma cell lines (U251 and U87) and normal human astrocytes (NHAs) were obtained from the American Type Culture Collection (ATCC, Manassas, VA, USA). All cell lines were cultured in Dulbecco’s Modified Eagle’s Medium (DMEM, Gibco, NY, USA) that was supplemented with 10% fetal bovine serum (FBS, Gibco), maintained at 37°C in a humidified incubator with 5% CO_2_.

Small interfering RNAs (siRNAs) targeting ADAMTS9-AS1 (si-ADAMTS9-AS1#1: 5′-ACAGCTATATCAGCCAACCAGAGT-3′′ and si-ADAMTS9-AS1#2: 5′-ACCAGCCAGAGCAAGCTATATACT-3′), as well as a negative control (si-NC: 5′-GTTTACAACACGCTTCCTCTGA-3′) were purchased from Santa Cruz Biotechnology Inc. (Dallas, TX, USA). U251 and U87 cells were plated in 6-well plates at a density of 1 × 105 cells per well and grown to reach 70-80% confluency, followed by a transfection with si-ADAMTS9-AS1#1, si-ADAMTS9-AS1#2, or si-NC for 48 h with lipofectamine 2000 Reagent (Invitrogen, Carlsbad, CA, USA) complied with manufacturer’s instruction.

### RNA extraction and quantitative real-time polymerase chain reaction (PCR)

Total RNA was extracted using the TRIzol RNA reagent (Invitrogen) following the manufacturer’s instructions. Reverse transcription was performed using cDNA synthesis kit (TaKaRa, Dalian, China). Quantitative real-time PCR was carried out on an ABI 7300 Fast Real-Time PCR system (Applied Biosystems, Foster City, CA) with SYBR Premix Ex Taq (Takara Biotech, Shiga, Japan), according to the thermocycling conditions: Initial denaturation at 95°C for 30 s followed by 40 cycles of extension at 95°C for 30 s and annealing at 60°C for 40 s. The primer sequences provided in this analysis were as follows: ADAMTS9-AS1, forward 5’-CCAGTTCGACCTTAGCTGCG-3’ and reverse 5’-CCAGAGCCCAGGGGTAAGAT-3’; GAPDH, forward 5’-ACGGATTTGGTCGTATTGGGCG-3’ and reverse 5’-GCTCCTGGAAGATGGTGATGGG-3’. Relative expression of ADAMTS9-AS1 was calculated by the 2-DDCT method.

### Cell Counting Kit-8 (CCK-8) assay

After 48 h transfection, cells were seeded into 96-well plates at a density of 4 × 10^3^ cells per well. Next, 10 μL of CCK-8 solution (Sigma-Aldrich, St. Louis, MO, USA) was added into cells in each well at 0, 24, 48, and 72 h, respectively. After another 2 h incubation, the absorbance at a wavelength of 450 nm was measured using a microplate reader (Bio-Tek, Instruments, Neufahrn, Germany).

### 5-ethynyl-2′-deoxyuridine (EdU) incorporation assay

The cell proliferation was assessed by performing EdU incorporation assay through the Cell-Light EdU DNA cell kit (RiboBio, China), in accordance with the manufacturer’s instructions. Briefly, transfected cells were seeded in 24-well plates. Then, 50 mM EdU labeling medium (Sigma-Aldrich) was loaded into the wells and incubated for 3 h at 37°C. Subsequently, 4% formaldehyde was dripped into each well for 20 minutes to fix the cells, followed by the addition of 1 × Apollo^®^ reaction cocktail (100 μL). After 30 minutes, DAPI was added to stain the nuclei and the percentage of Edu-positive cells was quantified in randomly selected three fields under fluorescence microscopy. Cells were fixed with 4% formaldehyde and permeabilized in 0.5% Triton X-100 for 20 minutes. Afterward, cells were incubated with 50 mM EdU (Sigma-Aldrich) for 3 h at 37°C and cell nuclei were stained with DAPI for 30 minutes. Finally, stained cells were observed under fluorescence microscopy and the percentage of Edu-positive cells was quantified in randomly selected three fields.

### Wound healing assay

For wound healing assay, transfected cells at a density of 2 × 10^5^ cells per well were seeded into 24-well plates and grown to 80% confluency. Then, a sterile 200 μl pipette tip was used to create linear scratch wounds. After being washed with PBS, cells were incubated for 24 h post-wound generation. The wound images were captured under a light microscope (×100) and the scratch area was assessed using Image J software (National Institutes of Health, Bethesda, MD, USA). Relative migration distance was calculated using the formula: (Area of 0 h wound – area of wound at 24 h)/area of 0 h wound × 100%.

### Transwell invasion assay

For invasion assay, transfected cells were suspended in 200 μl serum-free media and plated in the upper transwell chamber coated with 0.1 μl Matrigel (50 μg/ml, BD Biosciences, Franklin Lakes, NJ, USA). Meanwhile, a total of 500 μl complete media were added to the lower chamber as a chemoattractant. After the 24 hours incubation, we removed the cells on the upper chamber with cotton swabs and stained the cells that migrated into the lower chamber with 0.1% crystal violet for 30 minutes. The number of invasive cells was counted under a light microscope in randomly selected three fields.

### Western blot analysis

Total proteins were extracted using a radioimmunoprecipitation assay buffer with 1 mM phenylmethanesulfonyl fluoride (Sigma, St. Louis, MO, USA). After protein quantification through BCA protein assay kit (Beyotime), an equal amount of protein samples was separated by 10% sodium dodecyl sulfate/polyacrylamide gel electrophoresis and transferred to the PVDF membrane (Millipore, MA, USA). The membranes were blocked with Tris-buffered saline containing 0.1% Tween-20 (TBST) with 5% non-fat dry milk for 2 h. Subsequently, we performed incubation of primary antibodies against Wnt1, c-Myc, β-catenin, proliferating cell nuclear antigen (PCNA), E-cadherin, and GAPDH overnight at 4°C. After being washed with TBST for 5 times, the membranes were incubated with horseradish peroxidase-labeled secondary antibodies for 2 h at room temperature. Protein bands were visualized through enhanced chemiluminescence (Nanjing KeyGen Biotech Co., Ltd.) with GAPDH as the internal control.

### Statistical analysis

All *in vitro* experiments were performed in triplicate and repeated 3 times. Data were expressed as mean ± standard deviation (SD). All statistical analyses were performed using GraphPad Prism 6.0 software (GraphPad Software, San Diego, CA, USA). The association between ADAMTS9-AS1 expression and clinicopathological characteristics of glioma patients was analyzed using the Chi-squared test. The differences between patients with high or low levels of ADAMTS9-AS1 expression were investigated using Kaplan–Meier analysis with a log-rank test. Cox regression analysis was employed to perform univariate and multivariate analyses of survival data. The Student’s t-test or ANOVA test was applied to perform statistical differences analysis with *p* < 0.05 representing statistically significant.

## RESULTS

### Expression and prognostic potential of ADAMTS9-AS1 in glioma

**FIGURE 1:**
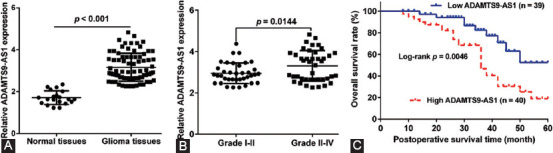
ADAMTS9-AS1 was upregulated in glioma tissues. (A) Relative expression of ADAMTS9-AS1 in glioma tissues and normal tissues was analyzed by quantitative real-time PCR; (B) relative expression of ADAMTS9-AS1 in high-grade and low-grade glioma tissue specimens by quantitative real-time PCR analysis; (C) Kaplan–Meier overall survival was analyzed according to ADAMTS9-AS1 expression.

To clarify the role of ADAMTS9-AS1 in the progression and development of glioma, we first determined the expression levels of ADAMTS9-AS1 in glioma tissues and normal brain tissues using quantitative real-time PCR analysis. As shown in Figure 1A, the expression level of ADAMTS9-AS1 in glioma tissues was significantly upregulated compared with that in normal brain tissues. We further divided these tumor tissues into 37 Grades I–II and 42 Grades III–IV, and compared the expression level of ADAMTS9-AS1 between the two groups. As shown in Figure 1B, the expression level of ADAMTS9-AS1 was increased in high-grade tissue specimens, compared with low-grade tissue specimens (*p* = 0.0144). Next, we analyzed the correlation between ADAMTS9-AS1 expression and clinicopathological features of patients with glioma. Based on the median value as a cutoff value, glioma patients were classified into the high (n = 40) or the low ADAMTS9-AS1 expression groups (n = 39). As listed in Table 1, ADAMTS9-AS1 expression level was apparently correlated with tumor size (*p* = 0.005) and the WHO grade (*p* = 0.002), but not significantly associated with age, gender, KPS score, and tumor location. Moreover, Kaplan–Meier analysis revealed that patients with high ADAMTS9-AS1 expression exhibited poor overall survival in comparison to patients with low ADAMTS9-AS1 expression (Figure 1C). Furthermore, Cox univariate and multivariate analysis suggested that the level of ADAMTS9-AS1 could serve as an independent prognostic factor affecting the overall survival of glioma patients (Table 2).

**TABLE 2:**
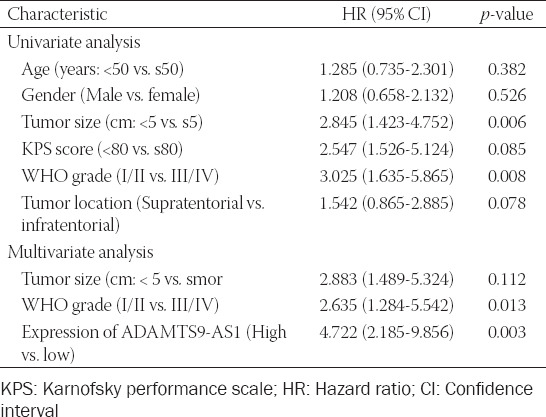
Univariate and multivariate analysis for overall survival in glioma patients

### Downregulation of ADAMTS9-AS1 played a negative role in regulating decreased cell proliferation in glioma cells

**FIGURE 2:**
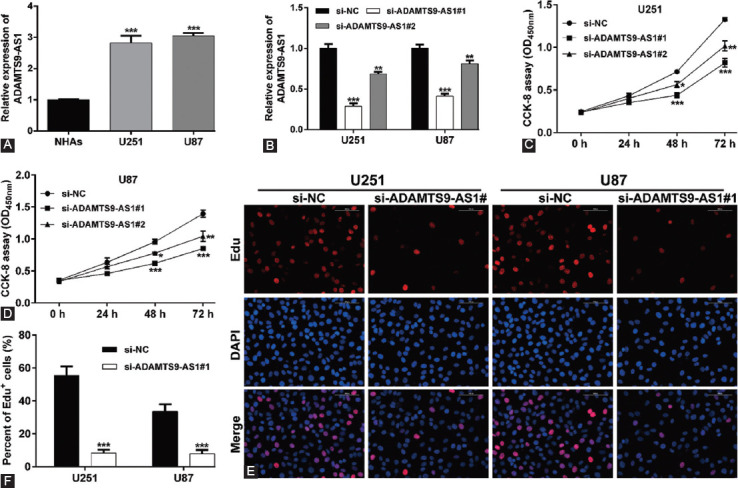
Knockdown of ADAMTS9-AS1 suppressed the proliferation of glioma cells. (A) The expression level of ADAMTS9-AS1 was assessed in normal human astrocytes (NHAs) and glioma cells (U251 and U87); (B) Transfection efficiency of si-ADAMTS9-AS1#1 or si-ADAMTS9-AS1#2 was determined by quantitative real-time PCR in U251 and U87 cells; (C-D) The cell viability of U251 and U87 cells transfected with si-ADAMTS9-AS1#1 or si-ADAMTS9-AS1#2 was evaluated using CCK-8 assay; (E) Edu staining was performed in U251 and U87 cells transfected with ADAMTS9-AS1#1 or si-ADAMTS9-AS1#2; (F) quantification of Edu-positive cells was performed in transfected U251 and U87 cells. Each experiment was performed in triplicate and measured for 3 times. *p < 0.05, **p < 0.01, and***p < 0.001 compared with si-NC.

To further confirm the functional roles of ADAMTS9-AS1 on glioma *in vitro*, we determined its expression in two glioma cell lines using quantitative real-time PCR analysis. As shown in Figure 2A, a significant increase of ADAMTS9-AS1 was revealed in glioma cells (U251 and U87) in comparison to NHAs. Subsequently, we knocked down ADAMTS9-AS1 expression in U251 and U87 cells by transfection with si-ADAMTS9-AS1#1 or si-ADAMTS9-AS1#2 (Figure 2B). CCK-8 assay showed that both si-ADAMTS9-AS1#1 and si-ADAMTS9-AS1#2 transfection significantly suppressed the cell viability in U251 (Figure 2C) and U87 (Figure 2D) cells, of which si-ADAMTS9-AS1#1 presented stronger suppressive effects on cell growth. Then, we selected si-ADAMTS9-AS1#1 as the subsequent analysis. According to the Edu incorporation assay, knockdown of ADAMTS9-AS1#1 remarkably suppressed cell proliferation in both U251 and U87 cells, as reflected by the decreased percentage of Edu-positive cells in si-ADAMTS9-AS1#1 group compared with si-NC group (Figure 2E and F).

### Downregulation of ADAMTS9-AS1 played a negative role in regulating decreased cell migration and invasion in glioma cells

**FIGURE 3:**
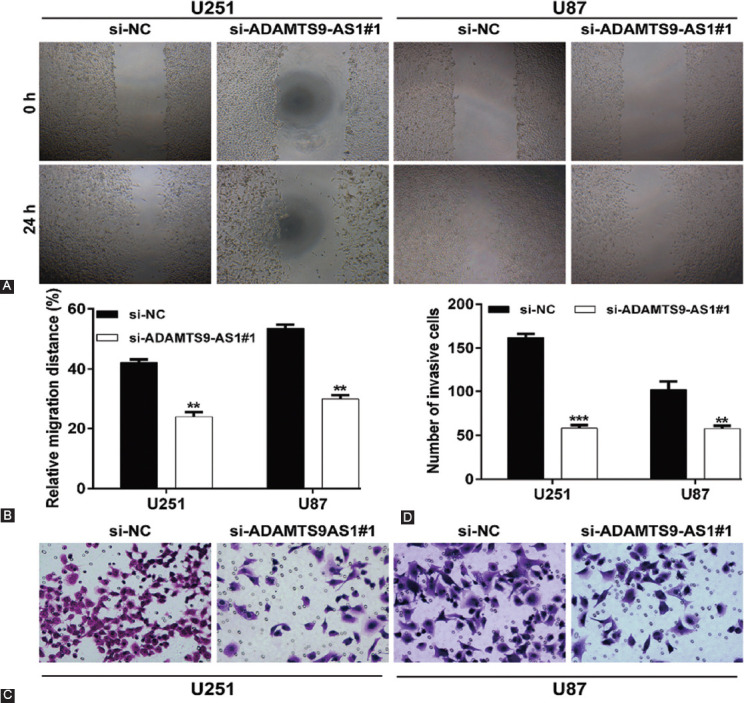
Knockdown of ADAMTS9-AS1 suppressed glioma cell migration and invasion. U251 and U87 cells were transfected with si-ADAMTS0-AS1#1 or si-NC, respectively. (A) Representative microscope images showing the filling of wound lines by wound healing assay in transfected U251 and U87 cells at 0 and 24 h; (B) quantification of relative migration distance after wound healing assay in transfected U251 and U87 cells; (C) representative microscope images showing the number of invasive cells by transwell invasion assay in transfected U251 and U87 cells; (D) quantification of invasive cells after transwell invasion assay in transfected U251 and U87 cells. Each experiment was performed in triplicate and measured for 3 times. **p < 0.01 and ***p < 0.001 compared with si-NC.

In addition to proliferation ability, we also analyzed the effects of ADAMTS9-AS1 on the motility ability of glioma cells. The results from the wound healing assay revealed that knockdown of ADAMTS9-AS1 significantly decreased the relative migration rate from 42.1% ± 1.1% to 23.9% ± 1.5% in U251 cells and from 53.5% ± 1.2% to 29.7% ± 1.3% in U87 cells (Figure 3A and B). Similarly, transwell invasion assay (Figure 3C and D) showed that the number of invasive cells was notably reduced in si-ADAMTS9-AS1#1 group compared with si-NC group in both U251 (si-ADAMTS9-AS1#1 vs. si-NC: 162.0 ± 4.0 vs. 58.3% ± 3.1) and U87 (si-ADAMTS9-AS1#1 vs. si-NC: 102.0 ± 10.0 vs. 57.7% ± 3.1) cells. In general, ADAMTS9-AS1 knockdown suppressed glioma cell migration and invasion.

### Downregulation of ADAMTS9-AS1 inhibited Wnt/β-catenin signaling pathway in glioma cells

**FIGURE 4:**
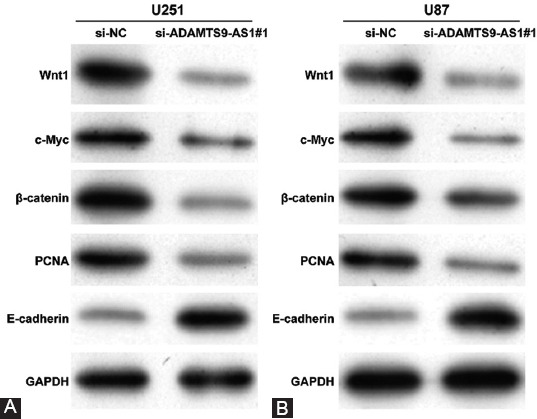
Downregulation of ADAMTS9-AS1 inhibited Wnt/β-catenin signaling pathway in glioma cells. The protein levels of Wnt1, c-myc, β-catenin, E-cadherin, and PCNA were detected in U251 (A) and U87 (B) cells after transfection with si-ADAMTS9-AS1#1 or si-NC.

Moreover, we addressed the molecular mechanisms underlying the ADAMTS9-AS1 knockdown suppressing glioma cell proliferation, migration, and invasion by investigating the activity of the Wnt/β-catenin signaling pathway. Using Western blot analysis, we observed that the expression of the nuclear Wnt1, c-myc, and β-catenin, which are the effectors of Wnt/β-catenin pathway, was obviously downregulated after the ADAMTS9-AS1 knockdown in U251 cells (Figure 4A). In addition, EMT marker protein E-cadherin was upregulated and proliferation-related protein PCNA was downregulated in U251 cells after transfection with si-ADAMTS9-AS1#1 compared with si-NC transfection. The same protein trends in the expression of Wnt1, c-myc, β-catenin, E-cadherin, and PCNA were also demonstrated in U87 cells following ADAMTS9-AS1 knockdown (Figure 4B).

## DISCUSSION

In this study, we confirmed that ADAMTS9-AS1 was overexpressed in glioma tissues and cell lines in comparison to the corresponding controls. By dividing 79 glioma patients into high and low ADAMTS9-AS1 expression group, we further demonstrated that ADAMTS9-AS1 expression level was correlated with tumor size and the WHO grade, which served as an independent prognostic factor affecting the overall survival of glioma patients. In line with our data, lower expression of ADAMTS9-AS1 represented a good prognosis in bladder urothelial carcinoma patients [26]. High ADAMTS9-AS1 level was associated with TNM stage, lymph node invasion, and worse survival prognosis in colorectal cancer patients [24]. Contrary to our results, lower expression levels of ADAMTS9-AS1 were observed in prostate cancer [22] and breast cancer tissues [19], which were identified as a novel prognostic biomarker for clinical application. The opposite expression levels of ADAMTS9-AS1 in tumor tissues were mainly ascribed to different tissue types, sample sizes, and different definitions of adjacent normal tissues.

To verify the function of ADAMTS9-AS1, the expression of ADAMTS9-AS1 was knocked down in U251 and U87 cells by transfection with si-ADAMTS9-AS1. After confirming the transfection efficiency, we further demonstrated that the knockdown of ADATMS9-AS1 significantly suppressed the proliferation, migration, and invasion abilities of U251 and U87 cells. In accordance with the present results, ADAMTS9-AS1 overexpression promoted the proliferation, migration, and invasion in MHCC97-H and HepG2 cells; ADAMTS9-AS1 knockdown showed the opposite results [23]. Chen et al. [24] reported that the depletion of ADAMTS9-AS1 significantly suppressed cell proliferation, G1/S transition, migration, and invasion in colorectal cancer. These findings suggested that ADAMTS9-AS1 played a positive role in glioma cell proliferation and metastasis *in vitro*.

Our data further elucidated the effects of ADAMTS9-AS1 on the molecular mechanisms underlying ADAMTS9-AS1 knockdown suppressing glioma cell proliferation, migration, and invasion. Typically, activation of the Wnt/β-catenin signaling pathway is considered to be one of the most important molecular pathways involved in the development of various human cancers, including glioma [27,28]. Moreover, inhibition of Wnt/β-catenin signaling can block the process of epithelial-mesenchymal transition (EMT) during the cancer progression [29]. Here, we demonstrated that downregulation of ADAMTS9-AS1 inhibited the protein levels of Wnt1, β-catenin, c-myc, and PCNA, while upregulating E-cadherin expression in both U251 and U87 cells. Our findings indicated the involvement of ADAMTS9-AS1 in the positive regulation of the Wnt/β-catenin signaling pathway in glioma, causing cell proliferation and metastasis *in vitro*. Notably, the regulatory role of ADAMTS9-AS1 on Wnt/β-catenin signaling has been elucidated in colorectal cancer by Li et al. who pointed that overexpression of ADAMTS9-AS1 could downregulate β-catenin and correspondingly decrease the downstream genes C-Myc and cyclin D1, which is the same as E-cadherin in colorectal cancer.

The different regulatory effects of ADAMTS9-AS1 on colorectal cancer and glioma might be limited to different tumor cells and experimental conditions. In addition, many lncRNAs, including PTCSC3 [27], NEAT1 [30], and BLACAT1 [31], have been reported to regulate Wnt/β-catenin signaling pathway to affect glioma cell growth, migration, and invasion. Thus, the results of the present study indicated that ADAMTS9-AS1 knockdown suppressed glioma cell functions, further affecting the Wnt/β-catenin signaling pathway. In addition, there were some limitations in our study as follows: (1) Lacking of publicly available datasets to perform *in silico* analysis and support these findings; (2) lacking *in vivo* experiments to validate the oncogenic role of ADAMTS9-AS1 in glioma; and (3) lacking of investigation on the effects of ADAMTS9-AS1 on drug resistance during glioma treatment; which will be our next study goal.

## CONCLUSION

Our findings suggested a crucial role of ADAMTS9-AS1 in the occurrence and development of glioma. These results indicated a potential clinical value of ADAMTS9-AS1 as a prognostic factor for the diagnosis of glioma. Importantly, ADAMTS9-AS1 enhanced cell proliferation, migration, and invasion in glioma by promoting the Wnt/β-catenin signaling pathway. Therefore, ADAMTS9-AS1 might be a novel therapeutic target for the treatment of glioma.
